# High polygenic risk score is a risk factor associated with colorectal cancer based on data from the UK Biobank

**DOI:** 10.1371/journal.pone.0295155

**Published:** 2023-11-30

**Authors:** Mei Yang, Vagheesh M. Narasimhan, F. Benjamin Zhan

**Affiliations:** 1 Department of Geography and Environmental Studies, Texas State University, San Marcos, Texas, United States of America; 2 Department of Integrative Biology, The University of Texas at Austin, Austin, Texas, United States of America; 3 Department of Statistics and Data Science, The University of Texas at Austin, Austin, Texas, United States of America; 4 Department of Population Health, University of Texas Dell Medical School, Austin, Texas, United States of America; National Cancer Institute, UNITED STATES

## Abstract

Colorectal cancer (CRC) is a common cancer among both men and women and is one of the leading causes of cancer death worldwide. It is important to identify risk factors that may be used to help reduce morbidity and mortality of the disease. We used a case-control study design to explore the association between CRC, polygenic risk scores (PRS), and other factors. We extracted data about 2,585 CRC cases and 9,362 controls from the UK Biobank, calculated the PRS for these cases and controls based on 140 single nucleotide polymorphisms, and performed logistic regression analyses for the 11,947 cases and controls, for an older group (ages 50+), and for a younger group (younger than 50). Five significant risk factors were identified when all 11,947 cases and controls were considered. These factors were, in descending order of the values of the adjusted odds ratios (aOR), high PRS (aOR: 2.70, CI: 2.27–3.19), male sex (aOR: 1.52, CI: 1.39–1.66), unemployment (aOR: 1.47, CI: 1.17–1.85), family history of CRC (aOR: 1.44, CI: 1.28–1.62), and age (aOR: 1.01, CI: 1.01–1.02). These five risk factors also remained significant in the older group. For the younger group, only high PRS (aOR: 2.87, CI: 1.65–5.00) and family history of CRC (aOR: 1.73, CI: 1.12–2.67) were significant risk factors. These findings indicate that genetic risk for the disease is a significant risk factor for CRC even after adjusting for family history. Additional studies are needed to examine this association using larger samples and different population groups.

## Introduction

Colorectal cancer (CRC) represents a significant global health challenge. It is the third most common malignancy and the second leading cause of cancer-related deaths worldwide [1, 2]. The burden of CRC is substantial, with approximately 1.8 million cases diagnosed globally in 2018 [3] and 1.93 million in 2020 [4]. Previous research has extensively explored the complexities of CRC, examining risk factors and mortality rates across various dimensions such as sociodemographic, socioeconomic, lifestyle, and geodemographic factors [5–13]. These studies have unveiled crucial insights into the disease. For example, research has identified a higher susceptibility to CRC in men [5, 6] and has linked socioeconomic deprivation to an elevated risk of emergency CRC diagnosis [7], particularly among young adults (20–39 years) with lower socioeconomic status [8]. Moreover, food availability and dietary choices have emerged as influential factors in CRC risk [9–11], while inadequate access to diagnosis and treatment services has significant consequences for the timing of CRC diagnosis and patient outcomes [12, 13]. This body of research collectively provides valuable insights into the multifaceted nature of CRC and its determinants, emphasizing the importance of comprehensive strategies for prevention and early intervention.

Although genetic factors are known to play an important role in the risk associated with CRC, genetic data were rarely combined with other factors in the analyses reported in the literature. The current study fills this research void by exploring the association between genetic factors and CRC risk, along with other relevant factors such as family history, sociodemographic, socioeconomic, and lifestyle factors. Additionally, most CRC studies reported in the literature focused on people aged 50 years or older [14–16]. Given that the CRC incidence rate in people younger than 50 has been trending up in the past few decades [2, 17–20], this study investigates risk factors associated with CRC for two separate age groups in the United Kingdom (UK): the older group (50+ years old) and the younger group, consisting of participants younger than 50.

Researchers have extensively utilized various datasets in their analysis of CRC, including the Genetics and Epidemiology of Colorectal Cancer Consortium, the Colon Cancer Family Registry, and the Colorectal Transdisciplinary Study [21–23]. However, prior investigation focused primarily on creating precise CRC risk prediction models, centering on diverse methods for constructing polygenic risk scores (PRS) for model comparisons or relied solely on PRS and composite environmental risk scores for CRC risk prediction. In contrast to these earlier studies, our research explicitly examines risk factors associated with CRC by including socio-environmental and lifestyle factors. Our objective is to identify specific factors that are closely correlated with CRC risk. In addition to genetic factors represented by PRS, we incorporate a broader spectrum of socio-environmental and lifestyle factors in our investigation, utilizing data from the UK Biobank.

## Materials and methods

### Data source

We used data from the UK Biobank, a population-based cohort study that collected blood samples from over 500,000 adults aged 40–70 years between 2006 and 2010, primarily across England, Scotland, and Wales. Samples underwent genotyping from blood derived cells using two arrays with a shared 95% marker content: the UK BiLEVE Axiom (UKBL; 807,411 markers) and the UK Biobank Axiom (UKBB; 825,927 markers). Genotype imputation was performed using reference panels from the Haplotype Reference Consortium, UK10K, and 1000 Genomes phase 3. In the biobank, 487,409 samples had imputed genotyping data available for this study. In addition to genetic data, the biobank also contains imaging data, health-related data, as well as sociodemographic and socioeconomic details for each participant. All participants were coded to protect their privacy.

In this study, we incorporated the following individual level data from the UK Biobank: family history, age, sex, body mass index (BMI), index of multiple deprivation (IMD), current tobacco smoking status, maternal smoking around birth, alcohol intake frequency, qualifications of education, current employment status, number of vehicles in the household, and average total house income. Family history of CRC, whether the father, mother, and siblings had CRC or not, is also available for this study. The units of BMI measurement were kg/m^2^. The IMD score was measured from seven distinct domains: income deprivation, employment deprivation, health deprivation and disability, education skills and training deprivation, barriers to housing and services, living environment deprivation, and crime.

The income deprivation domain examines income-related deprivation by counting individuals with low income across five indicators, such as those in income support families or receiving income-based Jobseeker’s allowances. The employment deprivation domain focuses on labor market exclusion, combining indicators like Jobseeker’s allowance claimants and Incapacity Benefit claimants. The health deprivation and disability domain evaluates premature mortality and reduced quality of life due to poor physical and mental health. The education skills and training deprivation domain assesses educational disadvantages for both children and adults. The barriers to housing and services domain considers geographical and financial obstacles to accessing housing and local services. The living environment deprivation domain evaluates indoor and outdoor living conditions. Lastly, the crime domain examines the recorded crime rate, including violence, burglary, theft, and criminal damage, as a reflection of personal and material victimization risk at a local level.

We dichotomized the variables based on their nature (Table 1). For example, participants were classified into those with a family history (father, mother, and siblings) of CRC (Yes) or not (No). Education level was categorized as either university or non-university education. The values of other categorical variables listed in Table 1 were coded in the same way. We exclusively included participants with complete records for all 13 variables listed in Table 1, and all 11,947 cases and controls included in the analysis had complete records.

**Table 1:** Summary information about the participants used in the study.

Variable	All cases and controls combined (%) (N = 11,947)	The older group (%) (2,387 cases and 8,579 controls) (N = 10,966)	The younger group (198 cases and 783 controls) (N = 981)
**Range of Age**	40–70	50–70	40–49
**Range of Body mass index (BMI)**	15.27–54.52	15.27–54.52	15.84–53.57
**Index of multiple deprivation (IMD)**	0.82–81.07	0.82–81.07	1.51–80.29
**Sex**			
Female	6,067 (50.8)	5,531 (50.4)	536 (54.6)
Male	5,880 (49.2)	5,435 (49.6)	445 (45.4)
**Polygenic risk score (PRS)**			
Low PRS	11,341 (94.9)	10,419 (95.0)	922 (94.0)
High PRS	606 (5.1)	547 (5.0)	59 (6.0)
**Family history**			
No	10,142 (84.9)	9,287 (84.7)	855 (87.2)
Yes	1,805 (15.1)	1,679 (15.3)	126 (12.8)
**Current tobacco smoking**			
No	11,059 (92.6)	10,183 (92.9)	876 (89.3)
Yes	888 (7.4)	783 (7.1)	105 (10.7)
**Alcohol intake frequency**			
Non-daily	8,879 (74.3)	8,065 (73.5)	814 (83.0)
Daily	3,068 (25.7)	2,901 (26.5)	167 (17.0)
**Household income**			
Above poverty line	9,644 (80.7)	8,746 (79.8)	898 (91.5)
Below poverty line	2,303 (19.3)	2,220 (20.2)	83 (8.5)
**Number vehicles in household**			
Have cars	11,294 (94.5)	10,368 (94.5)	926 (94.4)
No car	653 (5.5)	598 (5.5)	55 (5.6)
**Maternal smoking around birth**			
No	8,551 (71.6)	7,868 (71.7)	683 (69.6)
Yes	3,396 (28.4)	3,098 (28.3)	298 (30.4)
**Education**			
University	4,820 (40.3)	4,406 (40.2)	414 (42.2)
Non-university	7,127 (59.7)	6,560 (59.8)	567 (57.8)
**Employment**			
Employed	11,534 (96.5)	10,594 (96.6)	940 (95.8)
Unemployed	413 (3.5)	372 (3.4)	41 (4.2)

### Study subjects

We selected the CRC cases from the Biobank based on ICD-10 codes of C18.0-C18.9, C19, C20, and C26.0. Given that the majority of individuals in the genome-wide association study (GWAS) are of European ancestry, and considering the linkage disequilibrium (LD), allele frequency and gene-environment differences between populations, we included only samples of participants who are White British with complete imputed genotype information in the analyses [24]. Individuals with genetic relationships closer than the second degree were excluded (kinship coefficient > 0.0884). Controls were selected from the remaining 349,660 participants who are White British and were not diagnosed with CRC. Controls were randomly selected by matching cases within a 5-year age difference and with a residence location in the same output area (OA). Output areas are small geographic areas constructed using aggregation of postcode areas. The final dataset used in the analyses contained 2,585 cases and 9,362 controls.

Ethics approval was not required for this study because UK Biobank data is open to all researchers, and the data has been de-identified. We did not have access to any information that could identify individual participants during or after data collection.

### Polygenic risk score calculation

We calculated the PRS using 140 risk single nucleotide polymorphisms (SNPs) identified in a case-control study of CRC conducted by Thomas et al. [23]. This study used blood-derived genetic sequence information from all patients. The list of risk SNPs data and corresponding effect size on the risk of CRC can be found in the study of Thomas et al. [23]. One SNP in chromosome 13 (rs377429877) was missing in the imputed genotype data and was therefore excluded from the analyses. The SNPs in UK Biobank were imputed using the Haplotype Reference Consortium panel, with directly genotyped SNPs coded as 0, 1, or 2 copies of the risk allele, while imputed SNPs were coded as imputed dosages, indicating the anticipated number of risk allele copies. In general, we first extracted all the risk SNPs from the imputed genotyping data for each CRC case and control and then calculated the PRS as the sum of risk alleles of the respective variants (imputed dosages for imputed SNPs; 0, 1 or 2 copies of the risk alleles for genotyped SNPs). We used a scoring function in the PLINK 2.0 software [25] to calculate the PRS based on the imputed genotyping data in the UK Biobank. We followed the method used by Jia et al. and categorized individuals with a PRS in the top 5% in the high-risk group and other individuals in the low-risk group [26].

### Statistical analysis

We computed the odds ratios (OR) using logistic regression analysis based on a case-control study design for the cases and controls in the final dataset. The variables included in the analysis were age, BMI, IMD, sex, PRS, family history, current tobacco smoking, alcohol intake frequency, household income, number vehicles in the household, maternal smoking around birth, education, and employment. In addition, we divided the cases and controls into two groups: the older group, consisting of participants who were 50 years of age or older, and the younger group, who were less than 50 years old. The older group contained 2,387 cases and 8,579 controls and the younger group included 198 cases and 783 controls. We performed logistic regression analysis to compare the influence of the risk factors between these two age groups.

When performing the logistic regression analyses, we conducted univariate analysis to explore the impact of each variable on CRC individually. Additionally, we conducted multivariate analysis with all variables included in the model and compared the results with the univariate model. To examine how the results would differ when family history is excluded from the analysis and when only participants with top 5% and middle 41–60% PRS are considered, we conducted the same statistical analysis process on two sub-datasets selected from the current dataset: one comprising 10,142 participants without a family history of CRC, and the other consisting of participants with top 5% and middle 41–60% PRS.

## Results

Among 11,947 participants with complete data used in this study, more than half of them (6,067) were female (50.8%), especially in the younger group (54.6%). The BMI values ranged from 15.27 to 54.52, with a standard deviation of 4.5; the mean and median BMI were 27.2 and 26.7, respectively. The IMD values ranged from 0.82 to 81.07, with a standard deviation of 12.1; the mean and median IMD were 14.5 and 10.8, respectively. The older group had a slightly higher proportion of participants with less than a university education (59.8% vs 57.8%), a family history of CRC (15.3% vs 12.8%), and a significantly higher proportion of participants who drank daily (26.5% vs 17.0%) and had household incomes below the poverty level (20.2% vs 8.5%), compared to the younger group (Table 1). Conversely, the younger group had a slightly higher proportion of participants who were unemployed (4.2% vs 3.4%), with a high PRS (6.0% vs 5.0%), had maternal smoking around birth (30.4% vs 28.3%), and were active smokers (10.7% vs 7.1%), compared to the older group.

A brief examination of the data indicates that: (1) among the 2,585 CRC cases, 251 participants (9.7%) had a high PRS, and 2,334 participants (90.3%) had a low PRS; (2) among the 9,362 controls, 355 participants (3.8%) had a high PRS, and 9,007 participants (96.2%) had a low PRS. A higher proportion of participants with a high PRS was observed in the case group compared to that in the control group. A two-proportions z-test (α = 0.05) indicates that the difference between these two observed proportions is significant (p-value < 2.2 e-16).

Results of the analysis involving all 11,947 cases and controls revealed five significant risk factors. These five factors are, in descending order of the values of the adjusted odds ratios (aOR), high PRS (aOR: 2.70, CI: 2.27–3.19), male (aOR: 1.52, CI: 1.39–1.66), unemployment (aOR: 1.47, CI: 1.17–1.85), family history of CRC (aOR: 1.44, CI: 1.28–1.62), and age (aOR: 1.01, CI: 1.01–1.02) (Table 2). These five risk factors remain significant in the results of the analysis related to the older group and the ordering of the aOR values is the same, high PRS (aOR: 2.67, CI: 2.24–3.20), male (aOR: 1.56, CI: 1.42–1.72), unemployment (aOR: 1.49, CI: 1.17–1.89), family history of CRC (aOR: 1.43, CI: 1.26–1.61), and age (aOR: 1.02, CI: 1.01–1.03) (Table 3). For the younger group, only high PRS (aOR: 2.87, CI: 1.65–5.00) and family history of CRC (aOR: 1.73, CI: 1.12–2.67) are the two significant risk factors (Table 4).

**Table 2:** Results of logistic regression analysis: All cases and controls (2,585 cases and 9,362 controls).

Variables	OR (95% CI)	P value ^a^	aOR (95% CI)	P value ^b^
**Age**	**1.02 (1.01–1.02)**	**< 0.001**	**1.01 (1.01–1.02)**	**< 0.001**
**Body mass index (BMI)**	1.02 (1.01–1.03)	0.002	1.01 (0.99–1.02)	0.162
**Index of multiple deprivation (IMD)**	1.00 (0.99–1.01)	0.426	1.00 (0.99–1.00)	0.795
**Sex**				
Female	1 [Reference]	—	1 [Reference]	—
Male	**1.57 (1.44–1.72)**	**< 0.001**	**1.52 (1.39–1.66)**	**< 0.001**
**Polygenic risk score (PRS)**				
Low PRS	1 [Reference]	—	1 [Reference]	—
High PRS	**2.73 (2.31–3.23)**	**< 0.001**	**2.70 (2.27–3.19)**	**< 0.001**
**Family history**				
No	1 [Reference]	—	1 [Reference]	—
Yes	**1.51 (1.34–1.69)**	**< 0.001**	**1.44 (1.28–1.62)**	**< 0.001**
**Current tobacco smoking**				
No	1 [Reference]	—	1 [Reference]	—
Yes	0.99 (0.83–1.16)	0.856	0.93 (0.78–1.10)	0.387
**Alcohol intake frequency**				
Non-daily	1 [Reference]	—	1 [Reference]	—
Daily	1.14 (1.03–1.26)	0.009	1.06 (0.96–1.17)	0.258
**Household income**				
Above poverty line	1 [Reference]	—	1 [Reference]	—
Below poverty line	1.08 (0.97–1.20)	0.182	1.05 (0.93–1.19)	0.437
**Number vehicles in household**				
Have cars	1 [Reference]	—	1 [Reference]	—
No car	1.03 (0.85–1.24)	0.791	0.99 (0.80–1.21)	0.901
**Maternal smoking around birth**				
No	1 [Reference]	—	1 [Reference]	—
Yes	1.07 (0.98–1.18)	0.15	1.06 (0.96–1.17)	0.272
**Education**				
University	1 [Reference]	—	1 [Reference]	—
Non-university	1.05 (0.96–1.15)	0.259	1.03 (0.94–1.13)	0.494
**Employment**				
Employed	1 [Reference]	—	1 [Reference]	—
Unemployed	**1.54 (1.24–1.92)**	**< 0.001**	**1.47 (1.17–1.85)**	**0.001**

OR, Odds ratio; aOR, adjusted odds ratio;—, not applicable.

^a^ P value calculated by univariate logistic regression; significant at P < 0.05.

^b^ P value calculated by multivariate logistic regression; significant at P < 0.05.

* For the univariate regression model, only one variable was included in each model.

* For the multivariate regression model, all 13 variables listed in the table were included. These variables are age, BMI, IMD, sex, PRS, family history, current tobacco smoking, alcohol intake frequency, household income, number vehicles in the household, maternal smoking around birth, education, and employment.

**Table 3:** Results of logistic regression analysis: The older group (50 years or older; 2,387 cases and 8,579 controls).

Variables	OR (95% CI)	P value ^a^	aOR (95% CI)	P value ^b^
**Age**	**1.02 (1.01–1.03)**	**< 0.001**	**1.02 (1.01–1.03)**	**< 0.001**
**Body mass index (BMI)**	1.02 (1.01–1.03)	0.001	1.01 (0.99–1.02)	0.116
**Index of multiple deprivation (IMD)**	1.00 (0.99–1.01)	0.236	1.00 (0.99–1.01)	0.53
**Sex**				
Female	1 [Reference]	—	1 [Reference]	—
Male	**1.62 (1.47–1.77)**	**< 0.001**	**1.56 (1.42–1.72)**	**< 0.001**
**Polygenic risk score (PRS)**				
Low PRS	1 [Reference]	—	1 [Reference]	—
High PRS	**2.69 (2.26–3.21)**	**< 0.001**	**2.67 (2.24–3.20)**	**< 0.001**
**Family history**				
No	1 [Reference]	—	1 [Reference]	—
Yes	**1.48 (1.32–1.67)**	**< 0.001**	**1.43 (1.26–1.61)**	**< 0.001**
**Current tobacco smoking**				
No	1 [Reference]	—	1 [Reference]	—
Yes	1.05 (0.88–1.24)	0.617	0.98 (0.82–1.18)	0.86
**Alcohol intake frequency**				
Non-daily	1 [Reference]	—	1 [Reference]	—
Daily	1.13 (1.02–1.25)	0.022	1.05 (0.94–1.16)	0.417
**Household income**				
Above poverty line	1 [Reference]	—	1 [Reference]	—
Below poverty line	1.06 (0.95–1.19)	0.306	1.03 (0.91–1.17)	0.67
**Number vehicles in household**				
Have cars	1 [Reference]	—	1 [Reference]	—
No car	0.99 (0.82–1.22)	0.986	0.96 (0.78–1.19)	0.715
**Maternal smoking around birth**				
No	1 [Reference]	—	1 [Reference]	—
Yes	1.08 (0.98–1.19)	0.141	1.07 (0.96–1.18)	0.224
**Education**				
University	1 [Reference]	—	1 [Reference]	—
Non-university	1.07 (0.97–1.17)	0.185	1.04 (0.95–1.15)	0.417
**Employment**				
Employed	1 [Reference]	—	1 [Reference]	—
Unemployed	**1.53 (1.22–1.93)**	**< 0.001**	**1.49 (1.17–1.89)**	**0.001**

OR, Odds ratio; aOR, adjusted odds ratio;—, not applicable.

^a^ P value calculated by univariate logistic regression; significant at P < 0.05.

^b^ P value calculated by multivariate logistic regression; significant at P < 0.05.

* For the univariate regression model, only one variable was included in each model.

* For the multivariate regression model, all 13 variables listed in the table were included. These variables are age, BMI, IMD, sex, PRS, family history, current tobacco smoking, alcohol intake frequency, household income, number vehicles in the household, maternal smoking around birth, education, and employment.

**Table 4:** Results of logistic regression analysis: The younger group (<50 years old; 198 cases and 783 controls).

Variables	OR (95% CI)	P value ^a^	aOR (95% CI)	P value ^b^
**Age**	0.96 (0.90–1.02)	0.173	0.95 (0.90–1.01)	0.134
**Body mass index (BMI)**	0.99 (0.97–1.03)	0.943	0.99 (0.96–1.03)	0.942
**Index of multiple deprivation (IMD)**	0.99 (0.98–1.01)	0.443	0.99 (0.98–1.01)	0.405
**Sex**				
Female	1 [Reference]	—	1 [Reference]	—
Male	1.14 (0.84–1.56)	0.408	1.04 (0.75–1.45)	0.826
**Polygenic risk score (PRS)**				
Low PRS	1 [Reference]		1 [Reference]	
High PRS	**3.18 (1.85–5.48)**	**< 0.001**	**2.87 (1.65–5.00)**	**< 0.001**
**Family history**				
No	1 [Reference]	—	1 [Reference]	—
Yes	**1.79 (1.18–2.73)**	**0.006**	**1.73 (1.12–2.67)**	**0.014**
**Current tobacco smoking**				
No	1 [Reference]	—	1 [Reference]	—
Yes	0.58 (0.32–1.04)	0.067	0.52 (0.28–0.97)	0.04
**Alcohol intake frequency**				
Non-daily	1 [Reference]	—	1 [Reference]	—
Daily	1.31 (0.88–1.94)	0.184	1.36 (0.90–2.05)	0.144
**Household income**				
Above poverty line	1 [Reference]	—	1 [Reference]	—
Below poverty line	1.38 (0.82–2.32)	0.226	1.13 (0.59–2.18)	0.712
**Number vehicles in household**				
Have cars	1 [Reference]	—	1 [Reference]	—
No car	1.38 (0.74–2.58)	0.318	1.30 (0.62–2.74)	0.486
**Maternal smoking around birth**				
No	1 [Reference]	—	1 [Reference]	—
Yes	1.03 (0.73–1.44)	0.883	1.09 (0.76–1.54)	0.65
**Education**				
University	1 [Reference]	—	1 [Reference]	—
Non-university	0.92 (0.67–1.25)	0.58	0.90 (0.65–1.25)	0.525
**Employment**				
Employed	1 [Reference]	—	1 [Reference]	—
Unemployed	1.68 (0.84–3.35)	0.143	1.78 (0.80–3.92)	0.155

OR, Odds ratio; aOR, adjusted odds ratio;—, not applicable.

^a^ P value calculated by univariate logistic regression; significant at P < 0.05.

^b^ P value calculated by multivariate logistic regression; significant at P < 0.05.

* For the univariate regression model, only one variable was included in each model.

* For the multivariate regression model, all 13 variables listed in the table were included. These variables are age, BMI, IMD, sex, PRS, family history, current tobacco smoking, alcohol intake frequency, household income, number vehicles in the household, maternal smoking around birth, education, and employment.

To better understand the association between PRS and CRC, we analyzed a sub-dataset from the original case-control dataset, which included 10,142 participants without a family history of CRC. Among these participants, 9,287 (1,919 cases and 7,368 controls) are in the older group, and 855 (161 cases and 694 controls) are in the younger group. Tables 5 and 6 summarize the analysis results related to this sub-dataset. The results indicated that, for participants without family history of CRC, the risk for those with a high PRS to develop CRC is more than 2.90 times greater (aOR: 2.90, CI: 2.40–3.50) than those with a low PRS (Table 5). Age (aOR: 1.02, CI: 1.01–1.02), sex (aOR: 1.42, CI: 1.28–1.57), and employment status (aOR: 1.61, CI: 1.26–2.07) remained significant risk factors associated with CRC, consistent with the results in Table 2. There were slight changes in the OR compared to the analysis when family history was included as a factor. The risk of developing CRC is even higher for participants younger than 50 with a high PRS. It is 3.65 times greater (aOR: 3.65, CI: 1.95–6.84) (Table 6).

**Table 5:** Results of logistic regression analysis of the 10,142 participants without family history of CRC (2,080 cases and 8,062 controls).

Variables	OR (95% CI)	P value ^a^	aOR (95% CI)	P value ^b^
**Age**	1.02 (1.01–1.02)	< 0.001	1.02 (1.01–1.02)	< 0.001
**Body mass index (BMI)**	1.02 (1.01–1.03)	0.004	1.01 (0.99–1.02)	0.078
**Index of multiple deprivation (IMD)**	1.00 (0.99–1.01)	0.672	1.00 (0. 99–1.00)	0.914
**Sex**				
Female	1 [Reference]	—	1 [Reference]	—
Male	1.47 (1.33–1.62)	< 0.001	1.42 (1.28–1.57)	< 0.001
**Polygenic risk score (PRS)**				
Low PRS	1 [Reference]	—	1 [Reference]	—
High PRS	**2.86 (2.37–3.45)**	**< 0.001**	**2.90 (2.40–3.50)**	**< 0.001**
**Current tobacco smoking**				
No	1 [Reference]	—	1 [Reference]	—
Yes	0.99 (0.83–1.20)	0.976	0.92 (0.77–1.12)	0.413
**Alcohol intake frequency**				
Non-daily	1 [Reference]	—	1 [Reference]	—
Daily	1.17 (1.05–1.31)	0.004	1.11 (0.99–1.24)	0.082
**Household income**				
Above poverty line	1 [Reference]	—	1 [Reference]	—
Below poverty line	1.11 (0.98–1.25)	0.094	1.07 (0.93–1.22)	0.345
**Number vehicles in household**				
Have cars	1 [Reference]	—	1 [Reference]	—
No car	1.07 (0.87–1.31)	0.549	1.01 (0.81–1.26)	0.946
**Maternal smoking around birth**				
No	1 [Reference]	—	1 [Reference]	—
Yes	1.09 (0.98–1.21)	0.125	1.08 (0.97–1.20)	0.168
**Education**				
University	1 [Reference]	—	1 [Reference]	—
Non-university	1.03 (0.94–1.14)	0.531	1.02 (0.92–1.13)	0.743
**Employment**				
Employed	1 [Reference]	—	1 [Reference]	—
Unemployed	1.68 (1.33–2.13)	< 0.001	1.61 (1.26–2.07)	< 0.001

OR, Odds ratio; aOR, adjusted odds ratio;—, not applicable.

^a^ P value calculated by univariate logistic regression; significant at P < 0.05.

^b^ P value calculated by multivariate logistic regression; significant at P < 0.05.

* For the univariate regression model, only one variable was included in each model.

* For the multivariate regression model, all 12 variables listed in the table were included. These variables are age, BMI, IMD, sex, PRS, current tobacco smoking, alcohol intake frequency, household income, number vehicles in the household, maternal smoking around birth, education, and employment.

**Table 6:** Results of logistic regression analysis of the 855 participants without family history of CRC in the younger group (161 cases and 694 controls).

Variables	OR (95% CI)	P value ^a^	aOR (95% CI)	P value ^b^
**Age**	0.98 (0.92–1.05)	0.532	0.99 (0.92–1.06)	0.679
**Body mass index (BMI)**	0.99 (0.96–1.04)	0.957	0.99 (0.96–1.04)	0.933
**Index of multiple deprivation (IMD)**	0.99 (0.98–1.01)	0.492	0.99 (0.98–1.01)	0.326
**Sex**				
Female	1 [Reference]	—	1 [Reference]	—
Male	1.09 (0.77–1.54)	0.626	1.04 (0.72–1.49)	0.844
**Polygenic risk score (PRS)**				
Low PRS	1 [Reference]	—	1 [Reference]	—
High PRS	**4.01 (2.19–7.37)**	**< 0.001**	**3.65 (1.95–6.84)**	**< 0.001**
**Current tobacco smoking**				
No	1 [Reference]	—	1 [Reference]	—
Yes	0.60 (0.32–1.13)	0.114	0.52 (0.27–1.02)	0.056
**Alcohol intake frequency**				
Non-daily	1 [Reference]	—	1 [Reference]	—
Daily	1.35 (0.88–2.07)	0.172	1.36 (0.87–2.13)	0.178
**Household income**				
Above poverty line	1 [Reference]	—	1 [Reference]	—
Below poverty line	1.45 (0.80–2.63)	0.217	1.24 (0.60–2.58)	0.566
**Number vehicles in household**				
Have cars	1 [Reference]	—	1 [Reference]	—
No car	1.47 (0.75–2.90)	0.263	1.32 (0.59–2.96)	0.494
**Maternal smoking around birth**				
No	1 [Reference]	—	1 [Reference]	—
Yes	1.22 (0.85–1.76)	0.283	1.24 (0.85–1.81)	0.268
**Education**				
University	1 [Reference]	—	1 [Reference]	—
Non-university	1.01 (0.72–1.43)	0.95	0.98 (0.68–1.40)	0.895
**Employment**				
Employed	1 [Reference]	—	1 [Reference]	—
Unemployed	1.77 (0.83–3.77)	0.137	1.75 (0.75–4.11)	0.199

OR, Odds ratio; aOR, adjusted odds ratio;—, not applicable.

^a^ P value calculated by univariate logistic regression; significant at P < 0.05.

^b^ P value calculated by multivariate logistic regression; significant at P < 0.05.

* For the univariate regression model, only one variable was included in each model.

* For the multivariate regression model, all 12 variables listed in the table were included. These variables are age, BMI, IMD, sex, PRS, current tobacco smoking, alcohol intake frequency, household income, number vehicles in the household, maternal smoking around birth, education, and employment.

Furthermore, we analyzed a sub-dataset extracted from the original case-control dataset to compare it with prior findings, which included participants with a top 5% and middle 41–60% PRS, both with and without a family history of CRC. The sub-dataset that consisted of individuals with a family history of CRC comprised 2,988 participants, with 729 cases and 2,259 controls. The sub-dataset without a family history of CRC comprised 2,537 participants, with 590 cases and 1,947 controls. In the older group with a family history of CRC, there were 667 cases and 2,079 controls, whereas in the older group without a family history of CRC, there were 541 cases and 1,791 controls. Tables 7 and 8 summarize the outcomes for the selected participants at all ages. Supplementary tables (S1 and S2 Tables) summarize the outcomes for the selected older participants. The results demonstrate that individuals with a high PRS had two to three times greater risk (aOR: 2.86, CI: 2.36–3.47; aOR: 3.01, CI: 2.43–3.71) of developing CRC than those with a middle 41–60% PRS, regardless of their family history of CRC (Tables 7 and 8). These findings are consistent with previous results that categorized the PRS into high and low groups.

**Table 7:** Results of logistic regression analysis: All ages participants (729 cases and 2,259 controls).

Variables	OR (95% CI)	P value ^a^	aOR (95% CI)	P value ^b^
**Age**	1.01 (0.99–1.02)	0.195	1.01 **(**0.99–1.03**)**	0.072
**Body mass index (BMI)**	1.02 (0.99–1.04)	0.079	1.01 (0.99–1.03)	0.585
**Index of multiple deprivation (IMD)**	0.99 (0.99–1.01)	0.635	0.99 (0.99–1.01)	0.498
**Sex**				
Female	1 [Reference]	—	1 [Reference]	—
Male	**1.63 (1.38–1.93)**	**< 0.001**	**1.58 (1.33–1.89)**	**< 0.001**
**Polygenic risk score (PRS)**				
Low PRS (41–60%)	1 [Reference]	—	1 [Reference]	—
High PRS (top 5%)	**2.84 (2.35–3.44)**	**< 0.001**	**2.86 (2.36–3.47)**	**< 0.001**
**Family history**				
No	1 [Reference]	—	1 [Reference]	—
Yes	**1.42 (1.15–1.76)**	**0.001**	**1.31 (1.05–1.64)**	**0.017**
**Current tobacco smoking**				
No	1 [Reference]	—	1 [Reference]	—
Yes	0. 98 (0.72–1.35)	0.92	0.93 (0.67–1.30)	0.669
**Alcohol intake frequency**				
Non-daily	1 [Reference]	—	1 [Reference]	—
Daily	1.03 (0.85–1.24)	0.781	0.92 (0.76–1.13)	0.435
**Household income**				
Above poverty line	1 [Reference]	—	1 [Reference]	—
Below poverty line	0.97 (0.78–1.20)	0.764	0.98 (0.76–1.26)	0.86
**Number vehicles in household**				
Have cars	1 [Reference]	—	1 [Reference]	—
No car	0.96 (0.68–1.37)	0.836	0.95 (0.64–1.40)	0.777
**Maternal smoking around birth**				
No	1 [Reference]	—	1 [Reference]	—
Yes	1.03 (0.86–1.24)	0.754	1.04 (0.86–1.26)	0.669
**Education**				
University	1 [Reference]	—	1 [Reference]	—
Non-university	0.94 (0.79–1.11)	0.471	0.89 (0.75–1.07)	0.213
**Employment**				
Employed	1 [Reference]	—	1 [Reference]	—
Unemployed	**1.75 (1.16–2.62)**	**0.007**	**1.75 (1.13–2.70)**	**0.011**

OR, Odds ratio; aOR, adjusted odds ratio;—, not applicable.

^a^ P value calculated by univariate logistic regression; significant at P < 0.05.

^b^ P value calculated by multivariate logistic regression; significant at P < 0.05.

* For the univariate regression model, only one variable was included in each model.

* For the multivariate regression model, all 13 variables listed in the table were included. These variables are age, BMI, IMD, sex, PRS, family history, current tobacco smoking, alcohol intake frequency, household income, number vehicles in the household, maternal smoking around birth, education, and employment.

**Table 8:** Results of logistic regression analysis: All ages participants without family history of CRC (590 cases and 1,947 controls).

Variables	OR (95% CI)	P value ^a^	aOR (95% CI)	P value ^b^
**Age**	1.01 (0.99–1.02)	0.258	1.01 (0.99–1.03)	0.128
**Body mass index (BMI)**	1.01 (0.99–1.03)	0.178	1.01 (0.98–1.03)	0.62
**Index of multiple deprivation (IMD)**	0.99 (0.99–1.01)	0.647	0.99 (0.99–1.01)	0.538
**Sex**				
Female	1 [Reference]	—	1 [Reference]	—
Male	**1.55 (1.29–1.86)**	**< 0.001**	**1.53 (1.26–1.86)**	**< 0.001**
**Polygenic risk score (PRS)**				
Low PRS (41–60%)	1 [Reference]	—	1 [Reference]	—
High PRS (top 5%)	**2.92 (2.37–3.60)**	**< 0.001**	**3.01 (2.43–3.71)**	**< 0.001**
**Current tobacco smoking**				
No	1 [Reference]	—	1 [Reference]	—
Yes	0.90 (0.63–1.28)	0.543	0.82 (0.56–1.19)	0.292
**Alcohol intake frequency**				
Non-daily	1 [Reference]	—	1 [Reference]	—
Daily	1.06 (0.86–1.30)	0.611	0.97 (0.78–1.21)	0.77
**Household income**				
Above poverty line	1 [Reference]	—	1 [Reference]	—
Below poverty line	1.00 (0.79–1.28)	0.982	1.01 (0.77–1.34)	0.935
**Number vehicles in household**				
Have cars	1 [Reference]	—	1 [Reference]	—
No car	0.97 (0.66–1.45)	0.896	0.91 (0.59–1.40)	0.658
**Maternal smoking around birth**				
No	1 [Reference]	—	1 [Reference]	—
Yes	1.07 (0.87–1.31)	0.53	1.09 (0.88–1.34)	0.44
**Education**				
University	1 [Reference]	—	1 [Reference]	—
Non-university	0.91 (0.76–1.10)	0.325	0.88 (0.72–1.07)	0.202
**Employment**				
Employed	1 [Reference]	—	1 [Reference]	—
Unemployed	**1.75 (1.11–2.74)**	**0.016**	**1.73 (1.06–2.82)**	**0.027**

OR, Odds ratio; aOR, adjusted odds ratio;—, not applicable.

^a^ P value calculated by univariate logistic regression; significant at P < 0.05.

^b^ P value calculated by multivariate logistic regression; significant at P < 0.05.

* For the univariate regression model, only one variable was included in each model.

* For the multivariate regression model, all 12 variables listed in the table were included. These variables are age, BMI, IMD, sex, PRS, current tobacco smoking, alcohol intake frequency, household income, number vehicles in the household, maternal smoking around birth, education, and employment.

## Discussion and conclusion

Findings from this study suggest that a high PRS is a potential risk factor associated with CRC, regardless of whether individuals are older than 50 or younger. In addition, results from this study indicate that the risk for people younger than 50 with a PRS in the top 5% to develop CRC is 3.65 times greater than those whose PRS falls within the other 95%. This relative risk is higher than that for people without a family history of CRC compared to those with a family history of CRC. It is worth noting that high PRS had a higher odds ratio than family history of CRC based on the results of all logistic regression analyses. These findings have implications for the implementation of CRC screening programs aimed at preventing CRC or detecting it at an early stage. We suggest that additional research is needed to evaluate the findings, and we recommend that individuals with a high PRS should consider participating in CRC screening, even if they do not have a family history of CRC. In addition, our results demonstrate that while family history encompasses some form of genetic disease risk, having additional information from the PRS adds to risk stratification.

Previous studies have suggested that PRS is associated with CRC and has a stronger impact on early-onset CRC. Archambault et al. used 95 CRC-associated SNPs to study whether a PRS was associated with the risk of early-onset CRC [21]. Their results showed that PRS was significantly associated with early-onset CRC, and the association was stronger than CRC in people older than 50 years. Mur et al. weighed 92-variant-based PRS into 20 quantiles to assess the contribution of PRS to family history of CRC and early-onset CRC [27]. In their study, CRC patients in the highest weighted PRS quantile (the 20^th^ quantile), the top 5% weighted PRS, had a four-fold greater risk of developing CRC compared to those in the reference quantile (the 10^th^ quantile), the middle 46% -50% weighted PRS.

Jia et al. used risk variants to identify high-risk individuals for eight common cancers. The results showed that individuals with the highest 5% PRS had a two-to-three-fold elevated risk for developing CRC [26]. Ping et al. developed and validated PRS for CRC risk prediction in East Asians. Individuals within the top 5% of PRS had a 2.52-fold elevated CRC risk compared to those in the medium (41–60%) risk group [28]. Those results are consistent with the finding in this study that participants with a PRS in the top 5% had a two- or three times higher risk of developing CRC compared to those whose PRS is not in the top 5%.

Other previous studies have examined the association between PRS and CRC along with various risk factors, including lifestyle [29, 30], physical activity [31], consumption of red and processed meat [32], alcohol intake [33], smoking [34], frequency of colonoscopy [35, 36], and the use of non-steroidal anti-inflammatory drugs [37]. However, these previous studies primarily focused on PRS in isolation or in combination with just one additional relevant factor in their analyses. More comprehensive studies that incorporate PRS along with several risk factors are needed. Ibáñez-Sanz et al. developed a model to identify the CRC risk among Spanish population by using 21 CRC associated SNPs and incorporated environmental data such as lifestyle factors as well as family and medical history in their analysis [38]. The results from that study indicated that alcohol consumption, obesity, physical activity, red meat and vegetable consumption, and nonsteroidal anti-inflammatory drug use increased the risk of developing CRC. These researchers suggested that family history of CRC and risky SNPs are also factors leading to higher risk of developing CRC. These results support the findings from our study that participants with alcohol intake and a family history of CRC experienced an elevated risk of CRC. Although the study by Ibáñez-Sanz et al. considered impact of multiple factors on CRC, they simply counted the risk alleles across all 21 SNPs to represent the genetic risk. However, this approach has its limitations because it does not consider the effect sizes of SNPs. The PRS used in our study accounts for the effect sizes of SNPs.

Studies also evaluated whether a healthy lifestyle can offset increased genetic risk in CRC [39, 40]. Healthy lifestyle scores were constructed using numbers of lifestyle factors, and were categorized into unhealthy (unfavorable), intermediate, and healthy (favorable) groups. However, these studies considered lifestyle factors as a whole and it is not clear which exact underlying factor was associated with the development of CRC. Differs from these studies, we included multiple lifestyle factors as well as other factors in the analysis and explored their association with CRC individually instead of using a composite lifestyle score.

One strong aspect of our study is that it incorporates PRS with several other relevant factors compared to previous studies reported in the literature. These factors included sociodemographic, socioeconomic, lifestyle, and family history of CRC. Findings from this study hence fill some gaps in the literature. A limitation of our study is the small sample size for participants younger than 50 years old. The limited number of young participants in the study may account for the observed results, where only family history and PRS remain as statistically significant risk factors, while sex, employment status, and age lose their significance within the younger age group. Further investigation is warranted to validate these findings once a larger dataset of younger participants becomes available. The results are limited to British Whites only, as PRS calculations based on the identified risk SNPs from previous GWAS primarily involved individuals of European ancestry. Future research endeavors should examine whether the results would hold in other population groups based on large sample sizes.

## Supporting information

S1 Table Results of logistic regression analysis: The older group (50 years or older; 667 cases and 2,079 controls). (DOCX)

S2 Table Results of logistic regression analysis: The older group without family history of CRC (541 cases and 1,791 controls). (DOCX)
