# Supplementary material for: High polygenic risk score is a risk factor associated with colorectal cancer based on data from the UK Biobank
Source: PLoS One. 2023 Nov 30;18(11):e0295155. doi: 10.1371/journal.pone.0295155 (PMC10688735; doi:10.1371/journal.pone.0295155)
Supplement: S2 Table — (DOCX) [file pone.0295155.s002.docx]

**S2 Table. Results of logistic regression analysis: The older group without family history of CRC (541 cases and 1,791 controls).**

| **Variables** | **OR (95% CI)** | **P value ^a^** | **aOR (95% CI)** | **P value ^b^** |
| --- | --- | --- | --- | --- |
| **Age** | 1.02 (0.99 – 1.04) | 0.097 | 1.02 (1.00 – 1.04) | 0.053 |
| **Body mass index (BMI)** | 1.02 (0.99 – 1.04) | 0.148 | 1.01 (0.99 – 1.03) | 0.555 |
| **Index of multiple deprivation (IMD)** | 0.99 (0.99 – 1.01) | 0.833 | 0.99 (0.99 – 1.01) | 0.695 |
| **Sex** | | | | |
| Female | 1 [Reference] | — | 1 [Reference] | — |
| Male | **1.55 (1.27 – 1.88)** | **< 0.001** | **1.54 (1.25 – 1.89)** | **< 0.001** |
| **Polygenic risk score (PRS)** | | | | |
| Low PRS (41 – 60%) | 1 [Reference] | — | 1 [Reference] | — |
| High PRS (top 5%) | **2.84 (2.28 – 3.54)** | **< 0.001** | **2.92 (2.33 – 3.64)** | **< 0.001** |
| **Current tobacco smoking** | | | | |
| No | 1 [Reference] | — | 1 [Reference] | — |
| Yes | 0.95 (0.65 – 1.39) | 0.807 | 0.87 (0.59 – 1.30) | 0.503 |
| **Alcohol intake frequency** | | | | |
| Non-daily | 1 [Reference] | — | 1 [Reference] | — |
| Daily | 1.01 (0.82 – 1.26) | 0.903 | 0.92 (0.73 – 1.16) | 0.477 |
| **Household income** | | | | |
| Above poverty line | 1 [Reference] | — | 1 [Reference] | — |
| Below poverty line | 1.00 (0.78 – 1.28) | 0.992 | 1.00 (0.75 – 1.34) | 0.98 |
| **Number vehicles in household** | | | | |
| Have cars | 1 [Reference] | — | 1 [Reference] | — |
| No car | 1.01 (0.67 – 1.52) | 0.964 | 0.97 (0.62 – 1.52) | 0.886 |
| **Maternal smoking around birth** | | | | |
| No | 1 [Reference] | — | 1 [Reference] | — |
| Yes | 1.11 (0.89 – 1.37) | 0.355 | 1.13 (0.91 – 1.41) | 0.271 |
| **Education** | | | | |
| University | 1 [Reference] | — | 1 [Reference] | — |
| Non-university | 0.90 (0.74 – 1.10) | 0.303 | 0.87 (0.71 – 1.07) | 0.178 |
| **Employment** | | | | |
| Employed | 1 [Reference] | — | 1 [Reference] | — |
| Unemployed | 1.56 (0.96 – 2.53) | 0.072 | 1.55 (0.92 – 2.61) | 0.10 |

OR, Odds ratio; aOR, adjusted odds ratio; —, not applicable.

^a^ P value calculated by univariate logistic regression; significant at P < 0.05.

^b^ P value calculated by multivariate logistic regression; significant at P < 0.05.

* For the univariate regression model, only one variable was included in each model.

* For the multivariate regression model, all 12 variables listed in the table were included. These variables are age, BMI, IMD, sex, PRS, current tobacco smoking, alcohol intake frequency, household income, number vehicles in household, maternal smoking around birth, education, and employment.
